# Motivations of Older Climate Change Activists: Five Major Pathways to Engagement

**DOI:** 10.1093/geroni/igaf122.1266

**Published:** 2025-12-31

**Authors:** Haojing Zhu, Karl Pillemer

**Affiliations:** Cornell University, Ithaca, New York, United States; Cornell University, Ithaca, New York, United States

## Abstract

Mobilizing older environmental volunteers can provide a powerful solution to addressing both the environmental crisis and the aging society—two global challenges critical to achieving the United Nations’ sustainability development goals. Understanding how to motivate this demographic to engage in environmental volunteering is essential for harnessing the benefits of this solution. However, limited research directly addresses this topic. This study conducted semi-structured interviews with 54 older environmental activists to examine their motivations and patterns of engagement. Coding and analysis of the interviews identified five key motivational themes: 1) problem-solving, involving movement from issue identification to action; 2) the desire to contribute and make an impact; 3) immediate personal considerations; 4) beliefs and values; and 5) emotional motivations. Key findings include: 1) a strong desire to expand knowledge and challenge oneself to learn new skills; 2) an emphasis on the quality of social relationships rather than using volunteering to combat loneliness; and 3) both positive and negative emotions about nature motivating action. Future directions for research are discussed, including the emotional regulation role of nature and motivational differences across various volunteer programs, modes, and intensities. Also presented are practical insights for organizations to design more effective volunteer programs and infrastructures, helping recruit and retain older volunteers for environmental and climate change action.

